# Hormone replacement therapy is associated with improved cognition and larger brain volumes in at-risk *APOE4* women: results from the European Prevention of Alzheimer’s Disease (EPAD) cohort

**DOI:** 10.1186/s13195-022-01121-5

**Published:** 2023-01-09

**Authors:** Rasha N. M. Saleh, Michael Hornberger, Craig W. Ritchie, Anne Marie Minihane

**Affiliations:** 1 Norwich Medical School, University of East Anglia, Norwich, UK; 2 Centre for Clinical Brain Sciences, University of Edinburgh, Edinburgh, UK

## Abstract

**Background:**

The risk of dementia is higher in women than men. The metabolic consequences of estrogen decline during menopause accelerate neuropathology in women. The use of hormone replacement therapy (HRT) in the prevention of cognitive decline has shown conflicting results. Here we investigate the modulating role of *APOE* genotype and age at HRT initiation on the heterogeneity in cognitive response to HRT.

**Methods:**

The analysis used baseline data from participants in the European Prevention of Alzheimer’s Dementia (EPAD) cohort (total *n*= 1906, women= 1178, 61.8%). Analysis of covariate (ANCOVA) models were employed to test the independent and interactive impact of *APOE* genotype and HRT on select cognitive tests, such as MMSE, RBANS, dot counting, Four Mountain Test (FMT), and the supermarket trolley test (SMT), together with volumes of the medial temporal lobe (MTL) regions by MRI. Multiple linear regression models were used to examine the impact of age of HRT initiation according to *APOE4* carrier status on these cognitive and MRI outcomes.

**Results:**

*APOE4* HRT users had the highest RBANS delayed memory index score (P-APOE*HRT interaction = 0.009) compared to *APOE4* non-users and to non-*APOE4* carriers, with 6–10% larger entorhinal (left) and amygdala (right and left) volumes (*P*-interaction= 0.002, 0.003, and 0.005 respectively). Earlier introduction of HRT was associated with larger right (standardized *β*= −0.555, *p*=0.035) and left hippocampal volumes (standardized *β*= −0.577, *p*=0.028) only in *APOE4* carriers.

**Conclusion:**

HRT introduction is associated with improved delayed memory and larger entorhinal and amygdala volumes in *APOE4* carriers only. This may represent an effective targeted strategy to mitigate the higher life-time risk of AD in this large at-risk population subgroup. Confirmation of findings in a fit for purpose RCT with prospective recruitment based on *APOE* genotype is needed to establish causality.

**Supplementary Information:**

The online version contains supplementary material available at 10.1186/s13195-022-01121-5.

## Introduction

More than two-thirds of Alzheimer’s disease (AD) patients are women [1, 2]. The recent 2022 Global Burden of Disease (GBD) report shows that the age-standardized dementia prevalence is higher in women (female-to-male ratio= 1.69 (1.64–1.73)) [3]. Thus, higher incident cases in women cannot simply be explained by greater life expectancy [2, 4, 5]. The neurophysiological impact of estrogen decline during menopause is emerging as the main aetiological basis for the higher prevalence of AD in females [6, 7]. Estrogen receptors are expressed throughout the brain, with estrogen regulating multiple physiological processes including neuronal synaptic plasticity, neuroinflammation, brain macronutrient utilization, DHA metabolism, and blood brain barrier (BBB) integrity [8–10].

Consequently, the use of hormone replacement therapy (HRT) during the menopausal transition and post-menopausal period is being considered as a strategy to mitigate cognitive decline. Early observation studies showed that oral estrogen may be protective against dementia [11], with a risk reduction of 34% in an early meta-analysis [12]. However, results of clinical trials have been inconsistent [13, 14] or even shown harmful effects [15]. For example, the Women’s Health Initiative Memory study (WHIMS), in women over the age of 65 years, showed that the use of oral estrogen alone (conjugated Equine Estrogen, CEE) or CEE plus medroxyprogesterone acetate (MPA) resulted in a 49% or 76% increased risk of dementia respectively [16]. A recent meta-analysis showed that the negative effect of HRT on global cognition has been predominately tested in those >60years. In this meta-analysis, only two studies recruited participants <60years, with one showing no impact of oral estrogen on global cognition and the second showing a positive impact of CEE plus MPA on global cognition [17]. Limited recent, mainly preclinical, data has identified *APOE* genotype and age of HRT initiation as potential modulators of the HRT cognitive response [7, 18, 19].

*APOE* genotype is the most important common genetic determinant of cognitive decline and AD risk. In Caucasians, a 3–4-fold and 12–15-fold increased risk of AD is evident in *APOE3/E4* and *APOE4/E4* relative to the wild-type *APOE3/E3* genotype with several years earlier age of onset [20]. A greater penetrance of an *APOE4* genotype in females, first reported in the early 90s [6, 21], is likely to be an important contributor to the higher AD rates in women [6]. This somewhat understudied association has been reiterated over the years [22–24]. More decline in MMSE, immediate and delayed memory scores was observed with an increasing number of *APOE4* alleles in women compared to men [25].

The other possible contributor of inconsistencies to the impact of HRT on AD risk is the timing of its initiation [26]. For cognition and AD risk, HRT intervention may be most beneficial if introduced before a certain threshold of neuronal damage accumulates [7], with potentially a ‘critical window’ where HRT can be neuroprotective [27, 28]. This critical window is likely to be during the transition to menopause, where gradual estrogen decline increases the brain liability to AD-related pathologies. In a UK BIOBANK analysis, despite showing that cumulative life-time estrogen exposure was associated with increased brain aging (measured by cortical thickness, and cortical and subcortical volumes), a subgroup analysis revealed that women who started HRT earlier had less apparent brain aging compared to later starters. Importantly, this effect of HRT timing was only evident in *APOE4* carriers [29], raising the notion that the interaction of APOE and HRT initiation might have a significant effect on brain health later in life. However, to date, there are no comprehensive analyses investigating *APOE* genotype and HRT interactions on multiple cognitive and MRI outcomes in humans, which the current study addresses.

Therefore, we hypothesize that HRT will have more cognitive benefits in *APOE4* compared to non-*APOE4* women, in particular when introduced early during menopausal transition. The independent and interactive effect of *APOE* genotype and HRT use on select cognitive function tests and medial temporal lobe–related MRI brain volumes was tested cross-sectionally in the European Prevention of Alzheimer’s Dementia (EPAD) cohort.

## Materials and methods

### Participants and study design of the EPAD cohort

The European Prevention of Alzheimer’s Dementia (EPAD) project started in 2015 [30]. Its main objectives were to develop longitudinal models over the entire course of AD prior to dementia clinical diagnosis. These datasets will help in setting up a proof-of-concept trial (PoC) for the secondary prevention of AD [31]. Participants were included if they were above 50 years, with no dementia diagnosis at baseline. They should not have had any medical or psychiatric illness that could potentially exclude them from a future PoC trial. Recruitment was carried out in ten European countries.

The current analysis was carried out on the EPAD-LCS-v.IMI dataset, released in October 2020 and accessed from the Workbench of the Alzheimer’s Disease Data Initiative (ADDI) Portal website: https://portal.addi.ad-datainitiative.org/. The baseline (V1) data from 1906 participants was included, with numbers varying according to the availability of cognitive test scores and neuroimaging data.

### Demographics, *APOE* genotype, and hormone replacement therapy (HRT) data

Participants’ sex, age, years of education, handedness, and marital status were downloaded and analyzed. Those with a Clinical Dementia Rating (CDR) scale score > 1.0 were excluded, as scores ≥ 1 are indicative of cognitive impairment [32].

*APOE* genotype data were categorized into a non-E4 group, which included *E2/E2*, *E2/E3*, and *E3/E3*, and the E4 group, which included *E3/E4* and *E4/E4*. *E2/E4* genotype was excluded from the analysis as it includes both the protective and risk alleles.

HRT treatment data was used for women who were current or previous users of estrogen alone (estradiol, estriol, and estrone preparations) or combined estrogen plus progestogens preparations (native progesterone or progestins), via both oral and transdermal routes of administration, and at different doses. Age at HRT initiation was calculated by subtracting the duration of HRT use from the age of the participant.

### Cognitive test

The cognitive tests analyzed were based on the recommendations of the EPAD scientific advisory group [33] and included the Mini-Mental State Examination (MMSE), Dot counting to evaluate verbal working memory [34], and the Repeatable Battery for the Assessment of Neuropsychological Status (RBANS) total score and its five main indices that score for attention, delayed memory, immediate memory, language, and visuo-construction. Items comprising each index were also analyzed where necessary [35]. The RBANS total score is taken as the primary outcome of EPAD [36] due to its ability to detect and track very early cases of cognitive decline [37]. The Four Mountain Test (FMT) was used to detect changes in the allocentric and orientation spatial memory [38]. The supermarket trolley virtual reality test (SMT) was used to test for changes in egocentric space [39].

### MRI brain volumetric parameters

MRI volumetric brain neuroimaging data were downloaded from the ADDI portal. The protocols used, quality controls, and data transfer are described in detail elsewhere [36]. Briefly, brain MRI scans were carried out using standardized acquisition protocols. Regional gray matter volumes were determined through the 3D-T1 weighted images using a segmentation process based on atlas propagation with the Learning Embeddings for Atlas Propagation (LEAP) framework [40]. The analysis focussed on the medial temporal lobe (MTL) as the main brain region regulating cognition and memory processing [41]. The MTL includes the hippocampus (right and left), parahippocampus (right and left), entorhinal cortex (right and left), and amygdala (right and left). A wider exploratory analysis for 20 different brain regions was carried out separately and presented in the supplemental document.

### Statistical analysis

All data files were processed using RStudio and the statistical analysis was conducted by SPSS version 28 (IBM SPSS Statistics for Windows, Version 28.0. Armonk, NY: IBM Corp). Variables were checked for normality; box cox transformation and z-scoring were performed when required. Regional MRI volumes were adjusted to whole brain volume before statistical analysis.

#### Demographic analysis

Age, years of education, marital status, handedness, and CDR scores were compared between non-*APOE4*s and *APOE4*s, and HRT users and non-users. Independent *t*-tests were used to determine differences in normally distributed variables (age, years of education, and age at HRT initiation), with the Mann-Whitney test used for skewed variables (duration of HRT use). Chi-square test was used for categorical variables (marital status, handedness, CDR, and cardiovascular medication use).

#### Analysis of the impact of *APOE* genotype and HRT on cognition and volumes of medial temporal lobe components

Analysis of covariate analysis (ANCOVA) was used to investigate the main and interactive effects of *APOE* genotype and HRT use, on cognition and MTL brain volumes. Multivariate ANCOVA (MANCOVA) was used on RBANS indices and MTL regions. HRT and *APOE* genotype were the independent variables, while select cognitive tests and regional brain volumes were the dependent variables. Adjustment of the brain volumes to the whole brain volume was carried out before statistical analysis. Age, years of education, marital status, handedness, and CDR were used as covariates in the ANCOVA model.

Correction for multiple comparisons was carried out for each of the eight MTL regions (8 × 3) using the Benjamini-Hochberg false discovery rate (FDR) method. Uncorrected *p*-values for other brain regions (*n*=23) are represented in the supplemental information (Supplemental Table 1). Similarly, FDR correction for multiple testing was carried out for the cognitive tests (10 tests × 3).

To measure the magnitude of HRT-use effect on cognitive tests and MRI brain regions in *APOE4* carriers versus non-carriers, an effect size analysis was carried out. Cohen’s *d* [42] was used to compare the mean values between HRT users and non-users within each genotype group using the following equation: ( Mean_HRT-_mean_no-HRT_)/ standard deviation. In addition, partial Eta squared was used to measure the proportion of variance and was reported from the between-subject effect of the ANCOVA model.

#### Regression analysis

Multiple linear regression was carried out to determine the association between the age of HRT initiation with cognition and MTL-specific areas as dependent variables. Model-1 covariates: Age + years of education + handedness + marital status + CDR. Model-2: Model 1 covariates + age of HRT initiation. Reported results are for model-2.

## Results

### Demographics of women of the EPAD study at baseline according to APOE genotype

There was no difference in age, marital status, handedness, CDR, number of HRT users, and duration of HRT use between the *APOE4* and non-*APOE4* groups. *APOE4* carriers had less formal education (*P*= 0.014) (Table 1).

**Table 1:** Select baseline characteristics in women according to *APOE* genotype status

	Non-E4 (***n***=675)	E4 (***n***=399)	Total (***n***=1074)	***P***_**APOE**_
**Age (mean, SD)**	65.2 (7.3)	65.1 (7.3)	65.1 (7.3)	NS
**Years of education (mean, SD)**	14.4 (3.8)	13.8 (3.6)	14.1 (3.7)	0.014
**Marital status (*n*, %)**				NS
Married/cohabiting	443 (65.6)	280 (70.0)	723 (67.5)	
Divorced	108 (16.0)	48 (12.0)	156 (14.3)	
Single	64 (9.5)	31 (7.8)	95 (8.5)	
Widowed	59 (8.7)	40 (10.0)	99 (9.6)	
**Handedness (right/left)**	614/46	371/17	985/63	NS
**CDR (*n*, %)**				NS
0.0	477 (71.6)	265 (68.5)	742 (70.2)	
0.5	189 (28.4)	122 (31.5)	337 (29.8)	
**HRT use**				NS
Yes/no	52/623	31/368	83/991	
Current/past users	46/6	30/1	76/7	
Duration in years (mean, SD)	7.2 (6.26)	8.0 (7.17)	7.5 (6.58)	
Age at HRT initiation (years)	56.2 (8.36)	56.5 (7.01)	56.4 (7.83)	
**Current CV medication (*n*, %)**	196 (29.0)	130 (32.5)	326 (30.4)	NS

*NS* non-significant, CDR Clinical Dementia Rating, *CV* cardiovascular

### Impact of *APOE* genotype and hormone replacement therapy (HRT) use on cognitive status in women

A trend towards an APOE*HRT interaction (P-interaction = 0.097) was evident for the total RBANS score which was significant for the RBANS delayed memory index (P-interaction = 0.009), with scores consistently higher in *APOE4* HRT users compared to all other groups (Table 2).

**Table 2:** Cognitive outcomes scores (mean±SEM) according to HRT use and *APOE4* genotype status

	Non-E4							E4							P_***APOE***_	P_***HRT***_	P_APOE*HRT_
	No-HRT	***n***	HRT	***n***	Total	***n***	***p***-HRT	No-HRT	***n***	HRT	***n***	Total	***n***	***p***-HRT			
**MMSE total score**	28.49 ±0.07	603	28.43 ±0.36	50	28.49 ±0.07	653	0.607	28.15 ±0.11	350	28.22 ±0.30	30	28.16 ±0.10	380	0.960	0.565	0.724	0.782
**Dot counting score**	16.60 ±0.22	389	17.05 ±1.06	32	16.62 ±0.22	421	0.726	16.24 ±0.30	235	17.44 ±0.71	21	16.32 ±0.29	256	0.848	0.953	0.942	0.710
**RBANS scores**																	
**RBANS total scale**	103.57 ±0.62	600	105.04 ±2.78	49	103.63 ±0.61	649	0.921	100.52 ±0.85	351	106.68 ±3.44	29	100.88 ±0.83	380	0.045	0.488	0.128	0.097
**RBANS attention index**	97.65 ±0.70	601	102.61 ±2.73	28	97.86 ±0.68	629	0.222	97.23 ±0.93	352	102.23 ±3.34	29	97.51 ±0.90	381	0.706	0.818	0.297	0.652
**RBANS delayed memory index**	102.09 ±0.59	602	102.07 ±2.46	28	102.09 ±0.58	630	0.757	98.29 ±0.85	352	108.37 ±2.79	29	98.85 ±0.81	381	**0.002**	0.695	0.027^a^	0.009^a^
**RBANS immediate memory index**	106.55 ±0.58	602	105.18 ±30	28	106.49 ±0.57	630	0.854	101.65 ±0.87	352	105.59 ±3.83	29	101.87 ±0.85	381	0.150	0.434	0.307	0.209
**RBANS language index**	100.10 ±0.47	602	100.79 ±2.61	28	100.13 ±0.47	630	0.752	99.30 ±0.69	353	101.50 ±2.84	29	99.42 ±0.67	382	0.303	0.399	0.536	0.311
**RBANS visuo-constructional index**	105.16 ±0.65	602	106.82 ±2.95	28	105.23 ±0.63	630	0.310	104.66 ±0.92	352	108.32 ±2.91	29	104.87 ±0.88	381	0.483	0.163	0.938	0.233
**FMT total score**	8.31 ±0.43	32	9.33 ±0.33	3	8.40 ±0.40	35	0.803	7.48 ±0.55	33	10.50 ±1.50	3	7.77 ±0.56	36	0.195	0.449	0.271	0.439
**SMT total score**	6.54 ±0.63	34	6.33 ±0.88	3	6.53 ±0.58	37	0.781	5.14 ±0.54	33	10.00 ±1.53	4	5.53 ±0.55	37	0.158	0.549	0.451	0.241

Mean **±** SEM of cognitive test scores stratified according to *APOE* genotype and HRT use. Significant *P* values for *APOE* genotype, HRT, and APOE*HRT are shown, using the ANCOVA model (MANCOVA for RBANS scores). *p*-HRT within each *APOE* genotype is calculated using the pairwise comparison of the estimated marginal mean with Bonferroni adjustment for multiple comparison. Age, years of education, marital status, handedness, and CDR were used as covariates. *HRT* hormone replacement therapy, *MMSE* Mini-Mental State Examination, *RBANS* Repeatable Battery for the Assessment of Neuropsychological Status. *FMT* four mountain test, *SMT* supermarket trolley test. *P*-significant <0.05. ^a^ insignificant after FDR correction for multiple comparison. Bold: significant after FDR correction

**Fig. 1:**
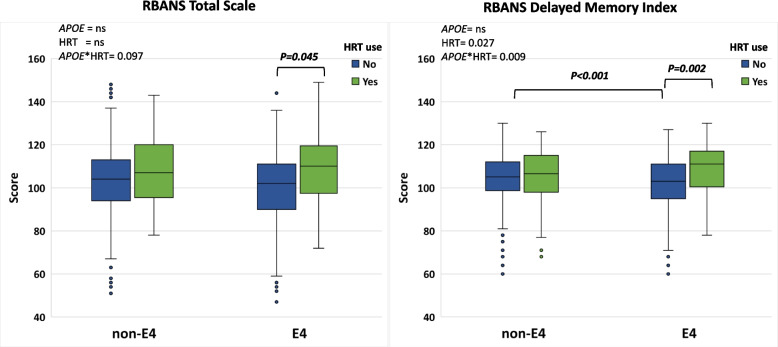
Box plots showing the mean scores of RBANS total scale (left) and RBANS delayed memory index (right) in non-*APOE4* versus *APOE4* stratified according to HRT use. Pairwise comparison within each genotype group was carried out on the estimated marginal mean (within the MANCOVA model), after adjustment for age, years of education, marital status, handedness, and CDR). Statistical results in the upper left corner show *P* values of *APOE* genotype, HRT, and APOE*HRT for RBANS total scale (left) and delayed memory index (right) using the MANCOVA model. Non-*APOE4 n*= 630 (no-HRT *n*=602, HRT *n*= 28), *APOE4 n*= 381 (no-HRT *n*=352, HRT *n*= 29)

Within *APOE* genotype group comparisons shows that HRT users have a higher RBANS total scale score (*p*= 0.045) and delayed memory index (*p*=0.002), only in *APOE4* carriers (Table 2 and Fig. 1). Effect size analyses (Cohen’s *d* and parial eta squared calculations) show a large effect of HRT use on FMT (Cohen’s *d*=0.988, parial eta squared = 0.08), and SMT (Cohen’s *d*=1.2, parial eta squared = 0.11) test scores. This large effect was found only in *APOE4 carriers*. Similarly, a moderate-to-large effect of HRT on the left entorhinal volume was observed in *APOE4 carriers* (Cohen’s *d* = 0.63, parial eta squared = 0.04) (Supplemental Table 2).

### Impact of *APOE* genotype and intake of hormone replacement therapy (HRT) on MRI volumes of medial temporal lobe–specific regions in women

Entorhinal (left) and amygdala (left and right) volumes were larger in *APOE4* HRT users compared to non-*APOE4* and non-HRT users (*P*-interaction= 0.002, 0.003, and 0.005, respectively) (Table 3). Similar trends were observed for the right entorhinal volume (*P*-interaction= 0.074). HRT users had larger left entorhinal (*P*= 0.03) smaller anterior cingulate gyrus (right and left, *p*= 0.003 and 0.062) and larger left superior frontal gyrus volumes (*p*= 0.009) than non-users, independent of their *APOE* genotype (Supplemental Table 1).

**Table 3:** MRI volumes (mean±SEM) of medial temporal lobe–specific regions, according to *APOE* genotype status and HRT use

	Non-E4				E4				P_***APOE***_	P_***HRT***_	P_APOE*HRT_
	No-HRT (***n***=591–593)	HRT (***n***=48–50)	Total (***n***=639–643)	***p***-HRT	No-HRT (***n***=345–347)	HRT (***n***=29)	Total (***n***=374–376)	***p***-HRT			
Right hippocampus	2360 ±13	2383 ±60	2362 ±13	0.367	2298 ±22	2384 ±37	2304 ±21	0.568	0.903	0.920	0.313
Left hippocampus	2284 ±13	2335 ±57	2288 ±12	0.690	2227 ±22	2333 ±43	2235 ±20	0.378	0.889	0.652	0.345
Right parahippocampal	2670 ±13	2735 ±50	2675 ±13	0.693	2624 ±20	2670 ±52	2628 ±19	0.370	0.430	0.660	0.966
left parahippocampal	2930 ±15	2971 ±46	2933 ±14	0.602	2840 ±20	2946 ±59	2849 ±19	0.801	0.587	0.698	0.302
Right entorhinal	2422 ±15	2444 ±53	2426 ±14	0.943	2378 ±22	2529 ±69	2373 ±22	0.040 ^a^	0.432	0.116	0.074
Left entorhinal	2026 ±12	2014 ±43	2029 ±12	0.426	1967 ±17	2172 ±55	1974 ±17	**<0.001**	0.106	0.026 ^a^	**0.002**
Right amygdala	1031 ±07	987 ±21	1029 ±06	0.030^a^	1002 ±11	1062 ±31	999 ±10	0.063	0.178	0.888	**0.005**
Left amygdala	1073 ±07	1038 ±23	1072 ±06	0.039^a^	1039 ±10	1105 ±33	1038 ±10	0.031 ^a^	0.204	0.661	**0.003**
Ventricular volume	25444 ±814	22262 ±1725	25201 ±763	0.949	24244 ±825	24265 ±3507	24245 ±805	0.860	0.987	0.858	0.920
Right cerebral WMV	10951 ±106	11287 ±351	10977 ±102	0.658	10697 ±140	10584 ±510	10688 ±135	0.291	0.908	0.266	0.576
Left cerebral WMV	11052 ±120	11408 ±372	11080 ±114	0.764	10847 ±146	10581 ±467	10826 ±140	0.961	0.630	0.886	0.842

Mean (mm^3^) ±SEM of medial temporal lobe–specific MRI brain volumes, according to *APOE4* genotype and HRT use. Significant *P* values for *APOE* genotype, HRT, and APOE*HRT are shown. MANCOVA model was used. *p*-HRT within each *APOE* genotype is calculated using the pairwise comparison of estimated marginal mean with Bonferroni adjustment for multiple comparison. Adjustment of the brain regions to the whole brain volume was carried out before statistical analysis. Age, years of education, marital status, handedness, and CDR were used as covariates. *p*-significant <0.05. ^a^ insignificant after FDR correction for multiple comparison. Bold: significant after FDR correction. *WMV* white matter volume

**Fig. 2:**
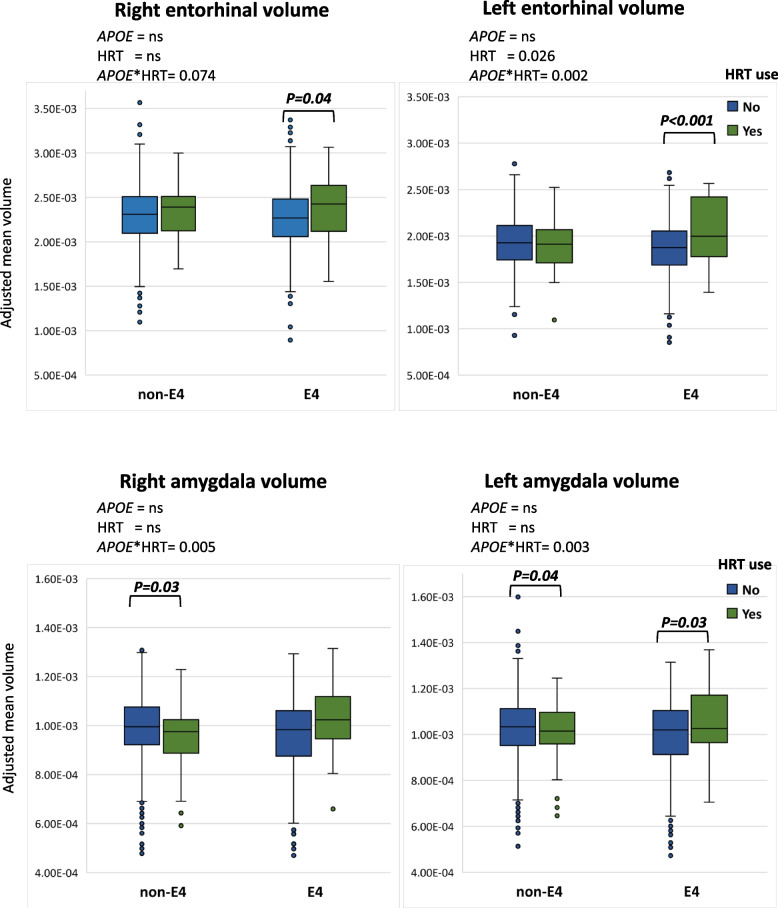
Boxplot showing the mean adjusted volumes of right and left entorhinal (up) and right and left amygdala volumes (down) in non-*APOE4* versus *APOE4* carriers, stratified according to HRT use. Pairwise comparison within each genotype group was carried out on the estimated marginal mean (within the MANCOVA model), after adjustment for age, years of education, marital status, handedness, and CDR. Volumes were adjusted to the whole brain volume*:* Statistical results in the upper left corner of the four brain regions show *P* values of *APOE* genotype, HRT, and APOE*HRT for each region using the MANCOVA model. Non-*APOE4 n*= 641 (no-HRT *n*=591, HRT *n*= 50), *APOE4 n*= 375 (no-HRT *n*=346, HRT *n*= 29)

Within *APOE* genotype group comparison shows that HRT users had larger entorhinal (right and left) and amygdala (left) volumes only in *APOE4* carriers. In non-*APOE4* carriers, amygdala volumes were smaller in HRT-users compared to non-users (Table 3 and Fig. 2).

### Earlier introduction of HRT was associated with larger hippocampal volumes only in *APOE4* carriers

**Fig. 3:**
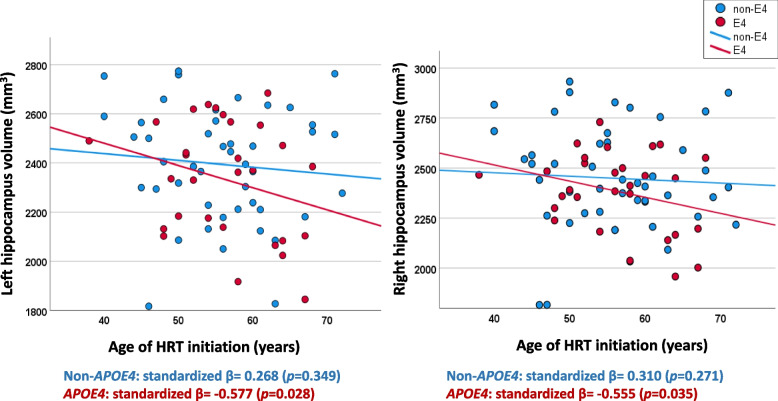
Association between right and left hippocampal volumes with age of HRT initiation. Multiple linear regression model showing the association between left and right hippocampal volumes and age of HRT initiation in non-*APOE4* carriers (blue) and APOE4 carriers (red). Model-1 covariates: Age + years of education + handedness + marital status + CDR. Model-2: Model 1 covariates + age of HRT initiation (as an independent variable). Reported results are of model-2. APOE4 *n*= 27. non-APOE4 *n*= 46

In *APOE4* carriers, a significant negative association between age of HRT initiation and hippocampal volumes was observed. Early HRT introduction was associated with larger right hippocampal volume (standardized *β*= −0.555, *p*=0.035) and left hippocampal volume (standardized *β*= −0.577, *p*=0.028). This association was not evident in non-*APOE4* carriers (Fig. 3). The association between right and left hippocampal volume and age of HRT initiation without stratification by *APOE* genotype was not significant (right hippocampus: standardized *β*= −0.086, *p*=0.601, left hippocampus: standardized β= −0.220, *p*=0.192). Associations of age of HRT initiation with entodhinal and amygdala volumes were not significant (Supplemental Fig. 1).

## Discussion

Higher AD risk and progression necessitates a more focused preventive approach in women. Results of the use of HRT for the prevention of cognitive decline have been inconsistent [14], or even harmful [15]. Here we demonstrate that *APOE* genotype and the age of HRT initiation are important modulators of the effect of HRT intervention on cognitive function and cognition-related brain volumes and can explain some of the discrepant outcomes. We report that *APOE4* women are most responsive to HRT. *APOE4* HRT users had larger entorhinal cortex and amygdala volumes compared to *APOE4*-non-HRT users and scored higher in the RBANS delayed memory index. We also importantly show that the earlier the age of HRT initiation, the larger the hippocampus volume, an association only observed in *APOE4* women. To our knowledge, this is the first study that demonstrates that HRT can have a beneficial effect on a range of cognitive function tests and cognition-related regional brain volumes in *APOE4* women.

In non-HRT users, *APOE4* carriers had a 3% lower delayed memory index score than non-*APOE4* (Table 2). This result is consistent with literature showing that delayed and immediate memory are lower in at-risk *APOE4* women [25, 43, 44]. HRT was found to influence cognition in an *APOE*-dependent manner. The use of HRT in *APOE4* women was associated with a 10% higher delayed memory scores. A recent meta-analysis of 23 RCTs showed no impact of oral estrogen alone or CEE plus MPA on delayed memory scores, even at a younger age (<60 years), with an overall negative effect on global cognition. However, no sub-group analysis according to *APOE* genotype was carried out [17].

There is limited evidence of a positive effect of HRT on cognitive tests and cognition-related brain region in *APOE4* carriers. In a sub-group analysis of the WHIMS RCT based on *APOE* genotype, a significant *APOE* × HRT interaction on the change in modified mini-mental status examination (3MSE) scores was observed, with the beneficial effect being exclusive to the homozygous *APOE4* carriers [45]. However, the analysis did not expand to include other cognitive tests or MRI findings [45]. In a UK BIOBANK analysis (age=40–69 years), early HRT use (oral and transdermal) was associated with less evident brain aging in *APOE4* carriers only [29]. In a small 2007 cross-sectional study, HRT use (*n*= 83) was associated with larger hippocampal volume in *APOE4* (*n*=15) compared to *APOE3* carriers [46].

Consistent with our cognitive data, *APOE4* HRT users had 6–10% larger right and left entorhinal and right and left amygdala volumes. Both the entorhinal cortex and amygdala play an important role in cognition, with the entorhinal cortex being one of the first regions to be affected by AD-related pathological changes, even before the hippocampus [47], independent of aging-related pathology [48, 49]. Consequently, reduction in entorhinal volume was considered as a better predictor than hippocampus volumes in the conversion from MCI to AD [50]. In *APOE4* carriers, smaller entorhinal volume in normal cognition [51] and with cognitive decline [52] have been observed. In the current study, no overall effect of *APOE* genotype on the entorhinal volume was evident. This is in line with previous studies which showed that at baseline, there were no differences in entorhinal volumes according to *APOE* genotype [53]; however, the rate of atrophy increases more with time in *APOE4* carriers, specifically in females [54].

Here we show that *APOE* genotype status influences the response to HRT, with larger entorhinal volume observed in *APOE4* carriers than non-carriers. The effect of HRT on cognition-related brain regions generally showed conflicting results [55, 56]. To our knowledge, only one study has showed that long-term HRT use was associated with larger hippocampus volume in *APOE4* carriers compared to *APOE3*, with associated higher N-acetylaspartate neuronal metabolic marker. However, no changes in cognitive test scores were observed [46]. In addition, in EPAD we also observed that HRT use (for an average of 8 years) is associated with a differential effect on the amygdala with higher volumes only in *APOE4* carriers.

Large effect of HRT use on FMT (Cohen’s *d*=0.988), and SMT (Cohen’s *d*=1.2) test scores were observed. These large effects were found only in *APOE4* carriers. Similarly, a moderate-to-large effect of HRT on the left entorhinal volume was observed in *APOE4* carriers (cohen *d* = 0.63). With the entorhinal cortex playing an important role in spatial navigation [57], and is affected in the early stages of AD pathology [47], one can deduce that the larger entorhinal volume could contribute to higher FMT and SMT scores in APOE4 HRT-users. Further research in human and experimental models are needed to confirm the strength of the association and causal relationships.

The amygdala plays an important role in cognition and emotion, which are inextricably linked [58]. In a recent meta-analysis, the volume of the hippocampus, parahippocampus, and amygdala was smaller in 2262 MCI patients compared to controls, [59]. Indeed, distinct amygdala radiomic features were able to distinguish between 97 AD patients, 53 MCI patients, and 45 normal controls, showing that microstructural changes in the amygdala can occur early during the course of AD [60]. The difference in amygdala volumes as a function of *APOE* genotype and HRT use is of interest, with a trend towards smaller volume in non-APOE4 HRT users. One could speculate that this might be due to the amygdala, as compared to other brain regions, being enriched in estrogen receptor (ER) alpha (ERα) but not ERβ [61]. This difference in enrichment may affect the structural and perfusion differences within this region [62]. This requires further future investigation.

An effect on cerebral blood flow (CBF) may explain the large entorhinal and amygdala volumes in *APOE4* HRT users. Previous studies show that *APOE4* carriers have higher CBF than non-carriers [63, 64]. This observation was seen at a younger age, with lower CBF observed in older age [65, 66]. HRT use in early menopause has been shown to improve peripheral vascular function [67], and in a recent study, estrogen use was associated with reduced cerebral vessels’ smooth muscle tone in a menopausal mouse model [68]. It is possible that a HRT-mediated effect on CBF in *APOE4* could in part underpin the impact on brain volume and associated improved memory, which is worthy of future investigation.

Another possible explanation for the select benefits in *APOE4* carriers is the complex interaction between *APOE4* genotype and age with neurophysiology and neuropathology. Although carrying the *APOE4* genotype increases the risk for cognitive decline and AD in older adults, select studies suggest that *APOE4* can be associated with enhanced cognitive performance at a younger age [69–71]. While the differential penetrance of *APOE4* by age is not well understood, it may be partly due to dysfunctional DHA metabolism [72, 73], β-amyloid clearance [74], or higher neuroinflammation [75] in older adults which all contribute to accelerated neuronal damage and loss, which may be mitigated by HRT use.

Besides *APOE* genotype, timing of HRT initiation is gaining attention as a mediator of the cognitive impact of HRT use, leading to the ‘critical window’ hypothesis [76]. This hypothesis suggests that the neuroprotective effects of HRT are only evident when it is introduced during the menopausal transition or early post-menopausal period [27]. Indeed, in the WHIMS RCT (age > 65 years), the use of oral CEE alone or CEE plus progestogen for up to 8 years was associated with 76% increased risk of dementia [16], and reduction in the hippocampus and total brain volumes [77]. However, there was no impact of HRT intervention on cognition in a younger subgroup (age 50-55 years, WHIMSY) (age 50–55) [78, 79]. Similarly, an observational study showed that the introduction of HRT in midlife (age 48.7 years) was associated with a 26% reduced risk of dementia compared to 48% increased risk when HRT was initiated in later life (age 76 years) [80]. In a recent meta-analysis in healthy post-menopausal women, HRT use was associated with reduced global cognition, which was less evident for those younger than 60 years old [17]. Our results are consistent with these findings, with our findings specifically observing that this impact of early initiation is specific to *APOE4* carriers. To our knowledge, the only study that looked at APOE*age of HRT initiation was carried out by De lange et al. [29], where less brain aging (calculated by machine learning) was associated with earlier HRT initiation in *APOE4* carriers.

### Limitations

This was a cross-sectional analysis precluding the establishment of causal relationship. Data about age of menopause, or if there was a gap between age at menopause and the start of HRT is not available, which would allow further granularity in our analysis. Information regarding the type of estrogen in the ERT formulation, and for example the use of conjugated estrogen versus estradiol was not available for some of the participants. In addition, doses of HRT were not available for some prescriptions. A further limitation is the small number of participants in the *APOE4* HRT user’s subgroup (*n*= 29–31) which did not allow for stratification according to the use of estrogen alone or estrogen plus progestogens preparation. The neurogenic effects of progestogen preparations are still controversial and require further investigations, with the available studies being mostly pre-clinical or carried out on small sample sizes [81–83].

## Conclusions

In conclusion, we show that HRT had more cognitive benefits in *APOE4* carriers. *APOE4* women on HRT performed better in delayed memory tasks and had large entorhinal and amygdala volumes than non-HRT users. Earlier HRT initiation was associated with larger hippocampus volume, an effect only seen in *APOE4* carriers. These results highlight the importance of personalized medicine in the prevention of AD. This work provides the rationale for the conduct of an RCT which examines the impact of early (perimenopause or early post-menopause) HRT introduction on cognition and brain atrophy according to *APOE4* carrier status to confirm the observed associations. Finally, the findings highlight the heterogenous nature of AD with the effectiveness of HRT in *APOE4* carriers only, indicating differences in the mechanistic basis of cognitive decline and pathology relative to non-carriers.

## Supplementary Information

**Additional file 1: Supplemental table 1.** Brain structural outcomes (MRI), volumes (mean±SEM) in mm^3^, according to HRT use and *APOE4* genotype status.

**Additional file 2: Supplemental table 2.** Effect sizes for the use of HRT on select cognitive test scores and regional brain volumes according to APOE genotype.

**Additional file 3: Supplemental figure 1.** Association between entorhinal (up) and amygdala volumes (down) with age of HRT initiation. Multiple linear regression model showing the association between entorhinal (left and right) and amygdala (left and right) volumes with age of HRT initiation in non-*APOE4* carriers (blue) and APOE4 carriers (red). Model-1 covariates: Age + years of education + handedness + marital status + CDR. Model-2: Model 1 covariates + age of HRT initiation (as an independent variable). Reported results are of model-2. APOE4 n= 27. non-APOE4 n= 46.

## Data Availability

The dataset is publicly available and can be accessed from the Workbench of the Alzheimer’s Disease Data Initiative (ADDI) portal website: https://portal.addi.ad-datainitiative.org/.
