# Supplementary material for: Hormone replacement therapy is associated with improved cognition and larger brain volumes in at-risk APOE4 women: results from the European Prevention of Alzheimer’s Disease (EPAD) cohort
Source: Alzheimers Res Ther. 2023 Jan 9;15:10. doi: 10.1186/s13195-022-01121-5 (PMC9830747; doi:10.1186/s13195-022-01121-5)
Supplement: Supplementary file 1 — Additional file 1: Supplemental table 1. Brain structural outcomes (MRI), volumes (mean±SEM) in mm3, according to HRT use and APOE4 genotype status. [file 13195_2022_1121_MOESM1_ESM.docx]

**Supplemental table 1:** **Brain structural outcomes (MRI), volumes (mean±SEM) in mm^3^, according to HRT use and *APOE4* genotype status**

|  | **Non-E4** | | | **E4** | | | | | **P*_APOE_*** | | | **P*_HRT_*** | | | **P*_APOE*HRT_*** |  |
| --- | --- | --- | --- | --- | --- | --- | --- | --- | --- | --- | --- | --- | --- | --- | --- | --- |
|  | **no-HRT (n=591-593)** | **HRT (n=48-50)** | **Total (n=639-643)** | | no-HRT**(n=345-347)** | HRT (n=29) | Total **(n=374-376)** |  | |  | | | |  | |  |
| Right hippocampus | 2360 ±13 | 2383 ±60 | 2362 ±13 | | 2298 ±22 | 2384 ±37 | 2304 ±21 | NS | | NS | | | | NS | |  |
| Left hippocampus | 2284 ±13 | 2335 ±57 | 2288 ±12 | | 2227 ±22 | 2333 ±43 | 2235 ±20 | NS | | NS | | | | NS | |  |
| right entorhinal | 2420 ±15 | 2494 ±48 | 2426 ±14 | | 2359 ±22 | 2540 ±81 | 2373 ±22 | NS | | 0.09 | | | | 0.074 | |  |
| Left entorhinal | 2026 ±12 | 2058 ±39 | 2029 ±12 | | 1957 ±17 | 2172 ±65 | 1974 ±17 | NS | | 0.026^a^ | | | | **0.002** | |  |
| Right amygdala | 1028 ±07 | 1032 ±21 | 1029 ±06 | | 993 ±11 | 1070 ±31 | 999 ±10 | NS | | NS | | | | **0.005** | |  |
| left amygdala | 1071 ±07 | 1082 ±19 | 1072 ±06 | | 1031 ±10 | 1116 ±33 | 1038 ±10 | NS | | NS | | | | **0.003** | |  |
| Right parahippocampal gyrus | 2670 ±13 | 2735 ±50 | 2675 ±13 | | 2624 ±20 | 2670 ±52 | 2628 ±19 | NS | | NS | | | | NS | |  |
| left parahippocampal gyrus | 2930 ±15 | 2971 ±46 | 2933 ±14 | | 2840 ±20 | 2946 ±59 | 2849 ±19 | NS | | NS | | | | NS | |  |
| Right Middle Temporal Gyrus | 6587 ±40 | 6831 ±14 | 6606 ±38 | | 6442 ±53 | 6635 ±185 | 6457 ±51 | NS | | NS | | | | NS | |  |
| left Middle Temporal Gyrus | 5250 ±30 | 5352 ±10 | 5258 ±29 | | 5154 ±38 | 5118 ±123 | 5151 ±36 | NS | | NS | | | | NS | |  |
| Right superior temporal gyrus | 6223 ±34 | 6391 ±12 | 6236 ±33 | | 6127 ±50 | 6154 ±190 | 6129 ±48 | NS | | NS | | | | NS | |  |
| left superior temporal gyrus | 6385 ±36 | 6435 ±12 | 6389 ±34 | | 6303 ±50 | 6256 ±148 | 6299 ±47 | NS | | NS | | | | NS | |  |
| Right anterior cingulate gyrus | 3543 ±25 | 3365 ±97 | 3529 ±24 | | 3564 ±33 | 3405±111 | 3552 ±32 | NS | | **0.003** | | | | NS | |  |
| left anterior cingulate gyrus | 4384 ±29 | 4286 ±85 | 4376 ±27 | | 4358 ±40 | 4350 ±133 | 4357 ±38 | NS | | 0.062 | | | | NS | |  |
| Right superior frontal gyrus | 12287 ±59 | 12580 ±17 | 12310 ±56 | | 12103 ±77 | 12001 ±275 | 12095 ±74 | 0.06 | | NS | | | | NS | |  |
| left superior frontal gyrus | 11582 ±57 | 12166 ±19 | 11627 ±55 | | 11472 ±77 | 11946 ±319 | 11509 ±75 | NS | | **0.009** | | | | NS | |  |
| Right medial frontal cortex | 1493 ±10 | 1497 ±41 | 1494 ±10 | | 1484 ±16 | 1457 ±42 | 1482 ±15 | NS | | NS | | | | NS | |  |
| left medial frontal cortex | 1675 ±11 | 1680 ±45 | 1676 ±11 | | 1632 ±15 | 1614 ±51 | 1631 ±15 | NS | | NS | | | | NS | |  |
| Right middle frontal gyrus | 16282 ±86 | 16101 ±21 | 16268 ±81 | | 16027 ±11 | 16446 ±27 | 16059 ±17 | NS | | NS | | | | 0.06 | |  |
| left middle frontal gyrus | 16497 ±88 | 16510 ±23 | 16498 ±83 | | 16267 ±12 | 16495 ±33 | 16285 ±11 | NS | | | NS | | NS | | | |

*Mean (mm^3^) ±SEM of regional MRI brain volumes, according to APOE4 genotype and HRT use***.** Significant P values for APOE genotype, HRT and APOE*HRT are shown. MANCOVA model was used. Adjustment of the brain regions to the total intracranial volume was carried out before statistical analysis. Age, years of education, marital status, handedness and CDR were used as covariates. p- significant <0.05. ^a^ insignificant after FDR correction for multiple comparison. **Bold**: significant after FDR correction
