# Supplementary material for: Hormone replacement therapy is associated with improved cognition and larger brain volumes in at-risk APOE4 women: results from the European Prevention of Alzheimer’s Disease (EPAD) cohort
Source: Alzheimers Res Ther. 2023 Jan 9;15:10. doi: 10.1186/s13195-022-01121-5 (PMC9830747; doi:10.1186/s13195-022-01121-5)
Supplement: Supplementary file 2 — Additional file 2: Supplemental table 2. Effect sizes for the use of HRT on select cognitive test scores and regional brain volumes according to APOE genotype. [file 13195_2022_1121_MOESM2_ESM.docx]

**Supplemental table 2: Effect sizes for the use of HRT on select cognitive test scores and regional brain volumes according to APOE genotype**

|  | **Non-E4** | | **E4** | |
| --- | --- | --- | --- | --- |
|  | **Cohen’s *d*** | **Partial Eta squared η^2^** | **Cohen’s *d*** | **Partial Eta squared** **η^2^** |
| **Cognitive tests** |  |  |  |  |
| **MMSE** | 0.124 (-0.159, 0 | 0.000 (0.161, 0.689) | 0.089 (-0.278, 0.456) | 0.000 (0.001, 0.975) |
| **Dot count** | 0.178 (-0.173, 0.528) | 0.000 (0.107, 0.743) | 0.116 (-0.320, 0.552) | 0.000 (0.105, 0.787) |
| **RBANS total scale** | 0.258 (-0.034, 0.549) | 0.000 (0.001, 0.977) | 0.457 (0.077, 0.837) | 0.009 (3.328, 0.069) |
| **FMT** | 0.456 (-0.727, 1.654) | 0.014 (0.388, 0.538) | **0.988 (-0.226, 2.188)** | **0.08 (2.604, 0.091)** |
| **SMT** | 0.061 (-1.241, 1.120) | 0.000(0.010, 0.920) | **1.202 (0.122, 2.267)** | **0.110 (3.9, 0.051)** |
|  |  |  |  |  |
| **Regional MRI volumes** |  |  |  |  |
| **Right hippocampus** | -0.080 (-0.383, 0.206) | 0.002 (1.096, 0.296) | 0.152 (-0.234, 0.537) | 0.001 (0.404, 0.526) |
| **Left hippocampus** | -0.003 (-0.300, 0.293) | 0.000 (0.185, 0.667) | 0.195 (-0.191, 0.580) | 0.002 (0.820, 0.366) |
| **Right parahippocampal** | 0.019 (-0.276, 0.313) | 0.000 (0.043, 0.836) | 0.074 ( -0.311, 0.459) | 0.001 (0.263, 0.609) |
| **left parahippocampal** | -0.067 (-0.361, 0.228) | 0.001 (0.320, 0.572) | 0.217 (-0.169, 0.602) | 0.004 (1.430, 0.233) |
| **right entorhinal** | 0.083 (-0.212, 0.376) | 0.000 (0.010, 0.950) | 0.389 (0.003, 0.775) | 0.013 (4.79, 0.029) |
| **Left entorhinal** | -0.027 (-0.321, 0.267) | 0.001 (0.420, 0.517) | **0.628 (0.289, 1.015)** | 0.038 (10.47, 0.001) |
| **Right amygdala** | -0.131 (-0.425, 0.164) | 0.005 (2.9, 0.085) | 0.371 (-0.015, 0.757) | 0.012 (3.9, 0.048) |
| **left amygdala** | -0.081 (-0.375, 0.213) | 0.003 (2.03, 0.155) | 0.426 (0.039, 0.812) | 0.014 (4.704,0.031) |

Cohen’s *d* effect sizes (95% confidence intervals) and partial eta-squared (F, significance). Cohen’s *d* of 0.2 indicates small effect; 0.5 indicates medium effect and 0.8 indicates large effect. Partial Eta Squared: η^2^ = 0.01 indicates a small effect; η^2^ = 0.06 indicates a medium effect; η^2^ = 0.14 indicates a large effect.
