# Supplementary material for: Hormone replacement therapy is associated with improved cognition and larger brain volumes in at-risk APOE4 women: results from the European Prevention of Alzheimer’s Disease (EPAD) cohort
Source: Alzheimers Res Ther. 2023 Jan 9;15:10. doi: 10.1186/s13195-022-01121-5 (PMC9830747; doi:10.1186/s13195-022-01121-5)
Supplement: Supplementary file 3 — Additional file 3: Supplemental figure 1. Association between entorhinal (up) and amygdala volumes (down) with age of HRT initiation. Multiple linear regression model showing the association between entorhinal (left and right) and amygdala (left and right) volumes with age of HRT initiation in non-APOE4 carriers (blue) and APOE4 carriers (red). Model-1 covariates: Age + years of education + handedness + marital status + CDR. Model-2: Model 1 covariates + age of HRT initiation (as an independent variable). Reported results are of model-2. APOE4 n= 27. non-APOE4 n= 46. [file 13195_2022_1121_MOESM3_ESM.pdf]

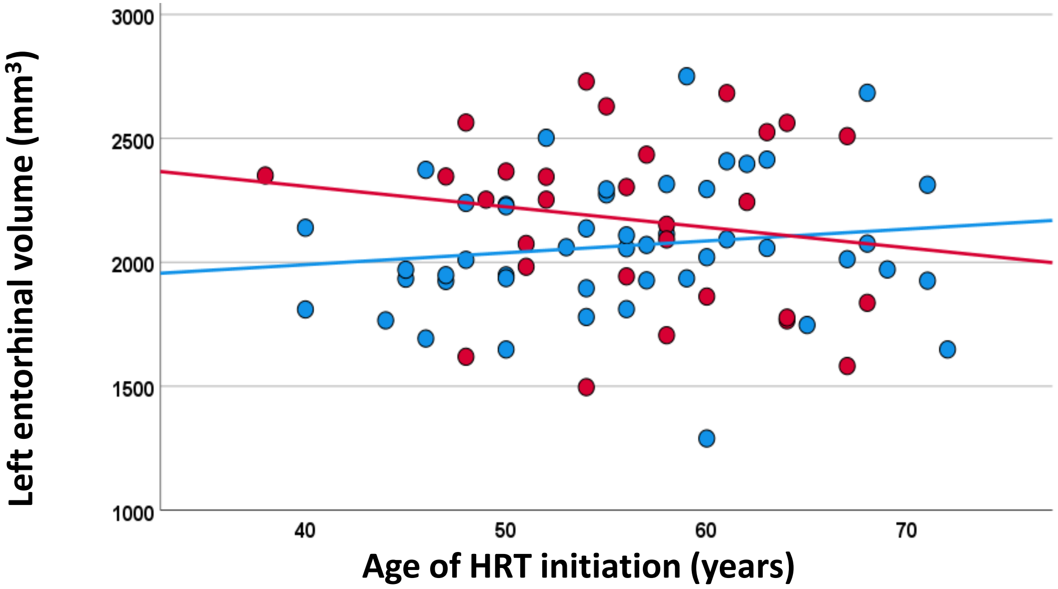

Non-*APOE4*: standardized β= 0.147 (p=0.193)
APOE4: standardized β= - 0.125 (p=0.710)

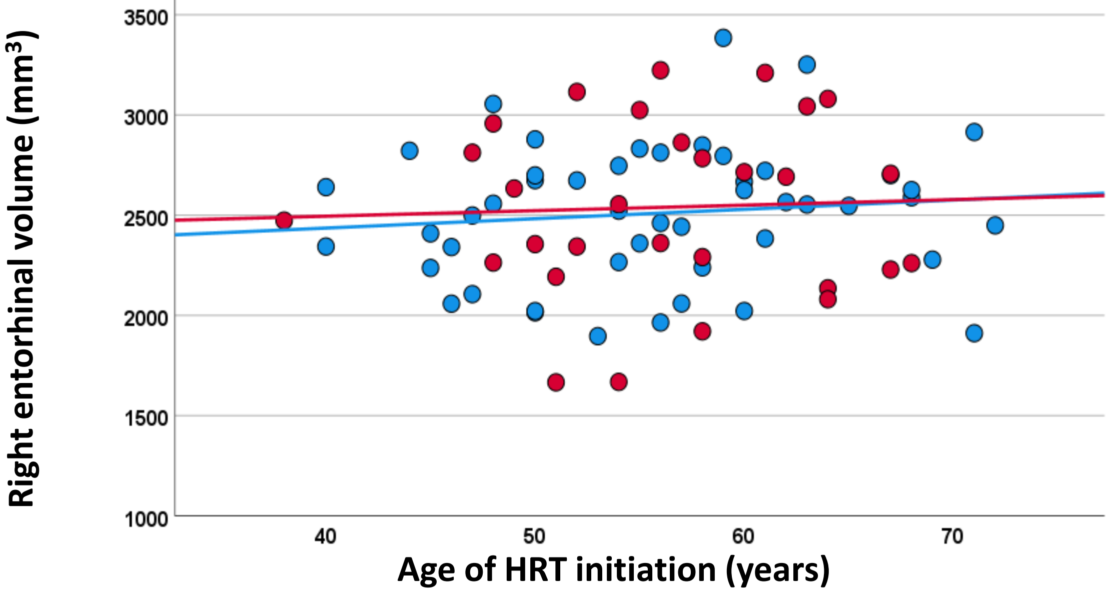

Non-*APOE4*: standardized β= 0.118(p=0.379)
APOE4: standardized β= 0.194(p=0.385)

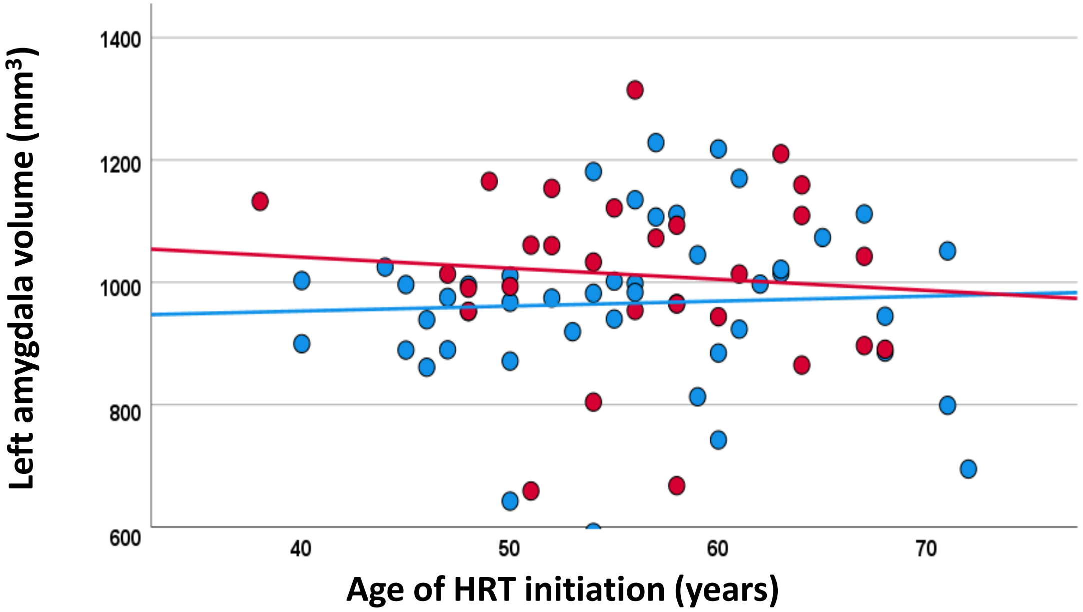

Non-*APOE4*: standardized β= 0.144(p= 0.506)
APOE4: standardized β= -0.025 (p=0.911)

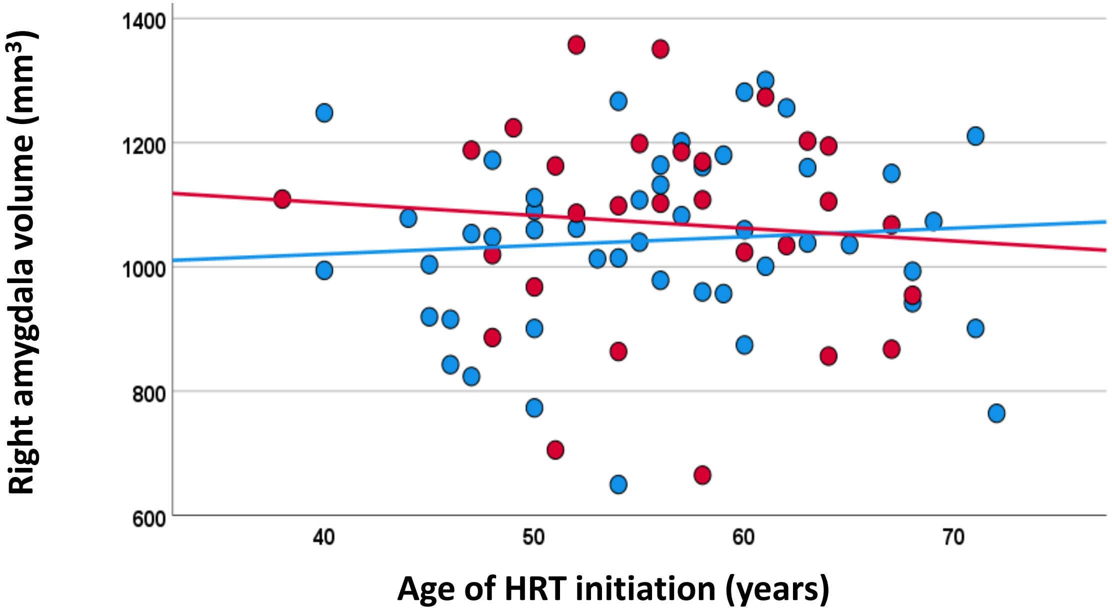

Non-*APOE4*: standardized β= 0.126 (p=0.406)
APOE4: standardized β= - 0.023(p=0.896)
